# Can Daytime Transcranial Direct Current Stimulation Treatment Change the Sleep Electroencephalogram Complexity of REM Sleep in Depressed Patients? A Double-Blinded, Randomized, Placebo-Controlled Trial

**DOI:** 10.3389/fpsyt.2022.851908

**Published:** 2022-05-18

**Authors:** Zhe Li, Xueli Zhao, Lingfang Feng, Yu Zhao, Wen Pan, Ying Liu, Ming Yin, Yan Yue, Xiaojia Fang, Guorui Liu, Shigeng Gao, Xiaobin Zhang, Norden Eh Huang, Xiangdong Du, Rui Chen

**Affiliations:** ^1^Sleep Center, The Second Affiliated Hospital of Soochow University, Suzhou, China; ^2^Sleep Center, Suzhou Psychiatric Hospital, The Affiliated Guangji Hospital of Soochow University, Suzhou, China; ^3^AidiSciTech Research Institute, Nanjing, China

**Keywords:** depression, electroencephalogram, intrinsic multi-scale entropy, rapid eye movement, transcranial direct current stimulation, randomized, double-blinded, placebo-controlled trial

## Abstract

**Study Objectives:**

The purpose of this study was to determine the effects of daytime transcranial direct current stimulation (tDCS) on sleep electroencephalogram (EEG) in patients with depression.

**Methods:**

The study was a double-blinded, randomized, controlled clinical trial. A total of 37 patients diagnosed with a major depression were recruited; 19 patients (13 females and 6 males mean age 44.79 ± 15.25 years) received tDCS active stimulation and 18 patients (9 females and 9 males; mean age 43.61 ± 11.89 years) received sham stimulation. Ten sessions of daytime tDCS were administered with the anode over F3 and the cathode over F4. Each session delivered a 2 mA current for 30 min per 10 working days. Hamilton-24 and Montgomery scales were used to assess the severity of depression, and polysomnography (PSG) was used to assess sleep structure and EEG complexity. Eight intrinsic mode functions (IMFs) were computed from each EEG signal in a channel. The sample entropy of the cumulative sum of the IMFs were computed to acquire high-dimensional multi-scale complexity information of EEG signals.

**Results:**

The complexity of Rapid Eye Movement (REM) EEG signals significantly decreased intrinsic multi-scale entropy (iMSE) (1.732 ± 0.057 vs. 1.605 ± 0.046, *P* = 0.0004 in the case of the C4 channel, IMF 1:4 and scale 7) after tDCS active stimulation. The complexity of the REM EEG signals significantly increased iMSE (1.464 ± 0.101 vs. 1.611 ± 0.085, *P* = 0.001 for C4 channel, IMF 1:4 and scale 7) after tDCS sham stimulation. There was no significant difference in the Hamilton-24 (*P* = 0.988), Montgomery scale score (*P* = 0.726), and sleep structure (N1% *P* = 0.383; N2% *P* = 0.716; N3% *P* = 0.772) between the two groups after treatment.

**Conclusion:**

Daytime tDCS changed the complexity of sleep in the REM stage, and presented as decreased intrinsic multi-scale entropy, while no changes in sleep structure occurred. This finding indicated that daytime tDCS may be an effective method to improve sleep quality in depressed patients. Trial registration This trial has been registered at the ClinicalTrials.gov (protocol ID: TCHIRB-10409114, in progress).

## Introduction

Major depressive disorder (MDD) is a common mental disorder with high recurrence and disability rates. Specifically, the annual and lifetime prevalence rates are as high as 6.6 and 16.2% (1). Recurrences leave patients with a heavy economic burden, a lower quality of life, and could be incremental (2). Between 50 and 90% of patients with depression complain about sleep disturbances (3). The symptoms of depression are complex and changeable, among which sleep disturbance is prominent, and early awakening is the characteristic sleep change of depression (4). Common sleep subjective characteristics in patients with depression include insomnia, light sleep, more dreams, easy awakening at night and so on. Polysomnography (PSG) is widely used to detect objective sleep structure in patients with depression. PSG research started in the 1960s with studies showing that major depression is characterized by alterations in sleep continuity (5). Because other sleep disturbances are common among other mental disorders, rapid eye movement (REM) sleep disturbances are considered a characteristic manifestation of depression disorders (6). In 1966 Hartmann et al. (7) reported that the REM sleep latency of patients with a depression disorder shortened at the beginning of sleep, while the proportion of REM increased. Some studies have shown that REM sleep in depressed patients tends to normalize after treatment (3, 8). A shortened REM sleep latency that exists after remission of depressive symptoms indicates that patients have a higher risk of relapse (8).

In the past, MDD patients have been mainly treated with pharmaco- and psycho-therapy (9). The shortcomings of pharmacotherapy include the lack of early onset response to treatment and side effects, which frequently cause treatment non-compliance (10). Non-invasive brain stimulation (NIBS) is increasingly used as an additive treatment for depression. Two major types of NIBS techniques are currently in use for clinical and research [transcranial magnetic stimulation (TMS) and transcranial direct current stimulation (tDCS)] (11). The latter technique, tDCS, delivers a weak current (1–2 mA) to the scalp for 10 min to regulate the membrane potential, which affects cortical activity and induces transient changes in brain function (12). Studies on tDCS and sleep have shown that using tDCS at night increases slow-wave sleep in healthy people (13). Studies have also shown that tDCS improves subjective sleep quality in college students (14). A subsequent analysis, however, revealed that tDCS treatment at night increases arousal in insomniacs (15). Existing studies have explored the efficacy of tDCS in the treatment of depression (16). tDCS is mostly treated in the daytime, with a frequency of five times a week, but few studies have explored the subjective and objective sleep quality at night (17–21).

An electroencephalogram (EEG) is a suitable option as a tool to investigate the brain. As a result, this method has been widely used for biomedical investigation (22, 23). The common methods of EEG signal analysis are linear and non-linear dynamic analyses. Studies have shown that non-linear EEG analysis can effectively explore the complexity of the human brain (24–26). Multi-scale entropy (MSE) is a typical non-linear approach, and the entropy of sleep EEG signals facilitates assessing the trajectory of brain maturation in newborns (27) and the characteristics of pathologic conditions, such as Parkinson’s disease (28). There are two significant drawbacks to MSE. MSE does not reflect the presence of high frequencies in the signal. MSE is not adapted to non-stationary or non-linear signals (29), which are unluckily the characteristics of sleep EEG signals. To overcome these drawbacks, intrinsic MSE (iMSE) was applied in this study.

Current studies have shown that even a single tDCS treatment lasts for at least 24 h (30). As a treatment, it is more practical to implement therapy during the day in the clinic. Thus, we sought to determine if use of tDCS during the daytime improve Sleep EEG complexity at night in depressed patients?

## Materials and Methods

### Study Design and Setting

**FIGURE 1:**
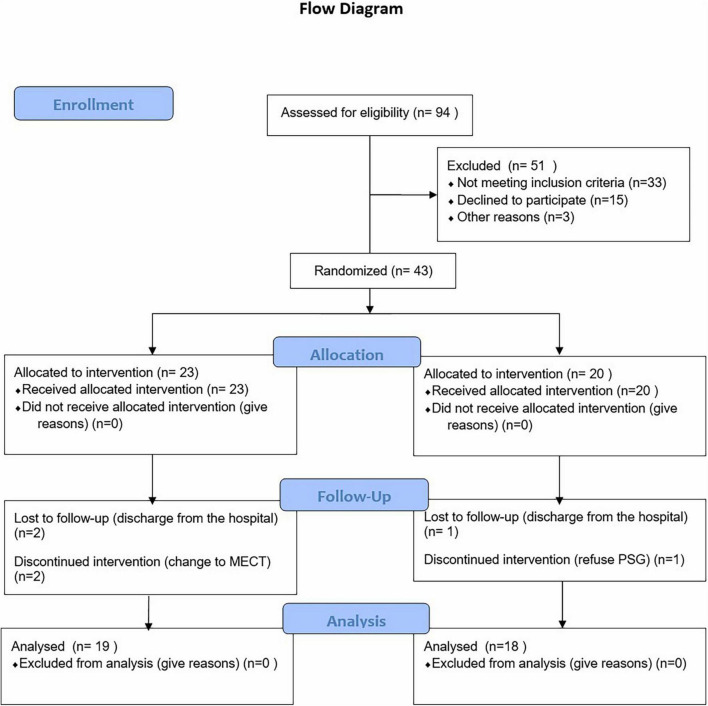
Flow diagram of the progress through the phases of a parallel randomized trial of two groups.

Full details of the study design, rationale, and methods as reported in compliance with CONSORT guidelines have been previously published (31). The CONSORT checklist is shown in Figure 1. The current study was a parallel, randomized, double-blinded, sham-controlled design with participants in an initial 2-week RCT phase. All participants completed the treatment during the daytime (five times per week for 2 weeks). The trial was registered and approved by the Ethics Committee of Suzhou Guangji Hospital (ClinicalTrials.gov ID: TCHIRB-10409114). All patients signed the informed consent.

### Participants

#### Inclusion and Exclusion Criteria

The inclusion criteria were as follows: (1) patients 20–65 years of age diagnosed with MDD according to DSM-5; (2) Hamilton Depression Rating scale-24 item (HDRS-24) score ≥20 with a third suicide factor score <3; (3) cognitive function sufficient to understand the research content and obtain informed consent; (4) escitalopram (20 mg/day) or duloxetine hydrochloride (60 mg/day) for 2 weeks; and (5) right-handed patients.

The exclusion criteria were as follows: (1) patients diagnosed with other psychiatric disorders, such as bipolar disorder or schizophrenia; (2) patients currently treated with modified electroconvulsive therapy (MECT) or rTMS within 6 months; (3) patients with severe medical and surgical diseases (epilepsy, dementia, craniocerebral injury, and severe liver dysfunction); and (4) recurrent headaches and skin allergies in the past.

### Randomize and Interventions

#### Randomization

First, patients admitted to the ward diagnosed with depression were evaluated, screened out according to the admission criteria, and informed consent was signed. Patients completed PSG and scale assessments at baseline. Then, after 2 weeks of treatment with escitalopram (20 mg/day) or duloxetine (60 mg/day), the patients were divided into active group or sham group according to a random counting table by the operating technician. After 10 tDCS treatments over a 2-week period, the patients were reassessed with a scale and PSG.

#### Interventions

Treatment was performed in a quiet environment with minimal communication between the therapist and the subject; the patient was not permitted to fall asleep. The anodes and cathodes were connected to 35-cm^2^ sponges soaked with 0.9% brine. According to the 10–20 system, F3 is the anode and F4 is the cathode.

The active tDCS group received 2-mA tDCS, and the current dose to the required parameter in 30 s. The stimulation lasted 20 min 5 times a week for a total of 10 stimulations. While the sham tDCS group had current for the first 10 s, and the current dropped to zero in 10–30 s, after which no current existed for the next 20 min. The sham button comes with the machine. The operator only needs to open the sham button.

### Measures

#### Measurement of Depression Severity

The severity of depression was measured at baseline and after tDCS using the Hamilton Depression Rating Scale (HDRS-24) and Montgomery scales (MARDS).

#### Polysomnography

Polysomnography is defined as the continuous monitoring and simultaneous recording of physiologic activity during sleep (32). PSG was used to record sleep EEG signals. The subjects were recorded while lying in an electromagnetic shielding chamber. Standard scalp electrodes were placed by the International 10–20 System; C3, C4, F3, F4, O1, and O2 referred to mastoid electrodes. The EEG was recorded at a 128-Hz sample frequency. Impedances were <5,000 ohms. Monitoring was performed using the SOMNOmedics V6 PSG system (company, city, Germany), with electrodes and sensors placed in a sleep diagnostic montage, as follows: six brain leads (F4 – M1, F3 – M2, C4 – M1, C3 – M2, O2 – M1, and O1 – M2); two eye movement leads (E1 – M2 and E2 – M2); 2 mandible muscle leads (CHIN1 – chinZ and CHIN2 – chinZ); left and right tibia anterior muscle conductance; and cardiac conductance. Subjects also wore oral and nasal heat sensors, nasal pressure sensors, RIP chest and abdomen breath sensing plethysmography tape, microphone snores sensor, a Nonin finger pulse oxygen saturation probe, and a posture sensor. Sleep technicians manually analyzed sleep and related events according to the AASM Manual for the Scoring of Sleep and Associated Events rules [version 2.2 (33)], then a sleep physician issued a report.

#### Intrinsic Multi-Scale Entropy

The sleep EEG signals were assessed by the iMSE method, which is quite suitable for non-linear and non-stationary EEG signals.

For each sleep stage of each participant, iMSE was calculated on eight parts of continuous 1,000 EEG data points (7.8125s). Artifacts, such as eye movements, blinks, muscle activities, or other artifacts, were excluded by independent component analysis (ICA). The artifacts were also visually checked.

The iMSE consists of two major parts: to compute the intrinsic mode functions (IMFs) of the sleep EEG signal; and compute the MSE of the cumulative sums of each of the IMFs. The IMFs were extracted with the empirical mode decomposition (EMD), which expanded a given time series into a set of narrowband oscillatory modes that emerged naturally from the inherent oscillatory modes within the signal (34). Those modes were termed IMFs and are data-driven.

In this study iMSE was applied to analyze sleep EEG signals of depressed patients before and after active/sham tDCS stimulation.

The calculation of iMSE was divided into two major steps EMD [i] and MSE calculation.

Part 1 Empirical mode decomposition.

An input signal.

$${\text{y}}_{0}\left(t\right),y_{0}\in R,t\in Zandt=\left[1:n\right]$$

was decomposed into a series of IMFs with EMD in the following process:

First, the upper and lower envelopes were acquired by connecting the local maxima and minima of the signal, respectively, with cubic splines.

Second, the average of the two envelopes was then removed from the original signal.

The sifting process (envelopes-acquiring and average-removing) was then repeated several times (usually 10 times). The first set of the sifting process obtained the first IMF, which carried higher frequencies than the residual signal with the first IMF removed.

Then, the residual signal was deemed as the input for a new round of iterations. In each sifting process turn, IMFs with lower frequencies were derived from the newly obtained residue of the last turn.

Finally, the result of the EMD was a decomposition of the signal [y_0_(*t*)] into the sum of the IMFs and a residue [r(t)]. That is,

$${\text{y}}_{0}\,\left(t\right)=\sum_{m=1}^{n_{m}}c_{m}\,\left(t\right)+r\,\left(t\right)$$

where *n*_*m*_ is the number of IMFs (35).

Part 2 MSE calculation.

1. The “multi-scale” of MSE was reflected in the process of coarse-graining, which was carried out in the following ways:

$$y_{j}^{\left(\tau \right)}=\frac{1}{\tau }\,\sum_{i=\left(j-1\right)\,\tau +1}^{j\,\tau }x_{i},1\leq j\leq N/\tau$$

For scale τ = 1, ${\text{y}}_{\text{j}}^{\left(1\right)}$ is the original signal and the length of the signal after coarse graining is *N*/τ.

2. Sample entropy was calculated for each coarse-grained time series. Sample entropy was calculated in the following way:

$$S\,a\,m\,p\,E\,n\,\left(m,r,N\right)=-\ln\frac{C^{m+1}\,\left(r\right)}{C^{m}\,\left(r\right)},$$

where *C*^*m*^(*r*) represents the ratio of sequence pairs the distance of which is <r and the whole sequence pairs after the sequence *u*(1),*u*(2),…,*u*(*N*) is divided into *N* − *m*+1 sequences, the length of which is *m* (36).

### Outcome Measures

The primary outcome was change in the iMSE and sleep structure of PSG over the 2-week RCT phase. Secondary measures were HDRS-24 total and factor scores.

### Numbers Analyzed

Due to the lack of previous work on iMSE-based clinical improvements before and after tDCS intervention in depression, the sample size calculation was not feasible. Instead, we surveyed similar work, and their sample sizes are 10 (37), 7 (38), 37 (39). Thus we planned to recruit at least 30 depressive patients for our study.

### Blinding

Patients and scale evaluators were blinded to group assignment. The technician covers the instrument during treatment so that the patient cannot see the treatment parameters.

## Statistical Analysis

The Chi-square test was performed to detect differences in primary physiological markers between the two groups. The mean ($\bar{x}$) and standard deviation (*s*) are expressed as $\bar{x}\pm s$ in tables.

A paired *t*-test or rank sum test were performed to detect differences in sleep structure and EEG complexity between pre- and post-treatment at every electrode site, every scale of MSE, and every IMF of EEG signals for active tDCS and sham tDCS groups. The alpha significance level was set at 0.05 and the 95% CI was also calculated.

The EEG complexity is acquired in multi-scale form, with different IMFs, scales of MSE, and electrode sites. Typically, the difference in EEG complexity before and after the stimulation is calculated in the case of channel C3, IMF 1:4, and MSE scale 7, the results of which are presented in the medians-quartiles form.

The sleep structure and HRDS-24 subscores were also analyzed using a two-sample *t*-test to detect early, middle, and late insomnia differences between active and sham groups.

## Results

Ninety-four patients admitted to the Sleep Department of Suzhou Guangji Hospital from October 2019 to December 2020 were evaluated. Among the patients, 33 did not meet inclusion criteria, 15 patients declined to participate, and 3 were discharged from the hospital without a cure after evaluation. Finally, 43 patients [active group (27) vs. sham group (24)] signed informed consent for the study, of whom 6 did not continue (Figure 1). In addition, patients were treated with monotherapy (escitalopram [20 mg/day] or duloxetine [60 mg/day]) 2 weeks after which the patients were randomly divided into active tDCS group (*n* = 23) and sham group (*n* = 20) using a random count table.

There were no statistically significant demographic or clinical characteristics differences (Table 1).

**TABLE 1:** Clinical characteristics of active and sham groups at baseline.

	Active group (*N* = 19)	Sham group (*N* = 18)	χ^2^	*P*
Sex, N (%)			1.3	0.25
Male	6 (31.58%)	9 (50%)		
Female	13 (68.42%)	9 (50%)		
Age	44.79 ± 15.25	43.61 ± 11.89	24.32	0.612
BMI	22.07 ± 3.04	23.19 ± 2.99	35	0.373
Baseline HRSD-24	19.42 ± 7.33	25.11 ± 6.43	19.99	0.395
Baseline MADRS	19.16 ± 8.98	24.83 ± 7.73	23.66	0.423

*There were no statistically significant demographic or clinical characteristics differences [mean ± S.D. (range)].*

**FIGURE 2:**
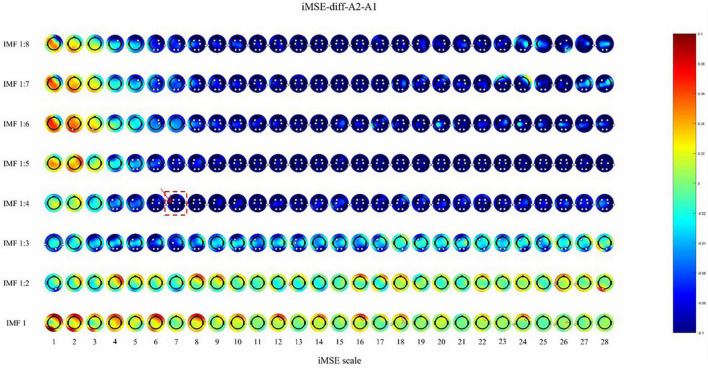
The difference before and after active stimulation in iMSE for REM stage. Negative values indicate decreased iMSE after stimulation. The small white dots indicate significant differences (*p* < 0.05). the color reflects the difference in the sleep EEG complexity before and after the stimulation. For example, the position where the color is blue, corresponding to –0.08 on the color bar, indicates that the EEG complexity decreased by 0.08 after stimulation. The white dots indicate that there is a significant difference (*p* < 0.05) in EEG complexity.

**FIGURE 3:**
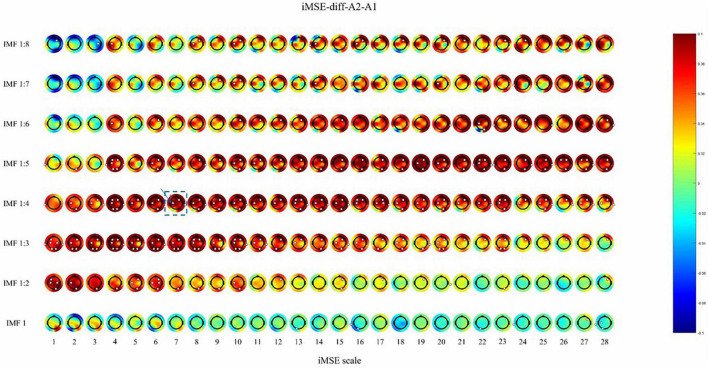
The difference before and after sham stimulation in iMSE for REM stage. Positive values indicate increased iMSE after stimulation. The small white dots indicate significant differences (*p* < 0.05).

After active stimulation there was a significantly decreased iMSE compared with pre-treatment for the REM stage (Figure 2). In addition, after sham stimulation there was a significantly increased iMSE compared with pretreatment (Figure 3).

### Recruitment

An interim analysis was performed because of slow accrual. We are still recruiting new subjects for later stratification analysis.

### Sleep Structure and Measuring Scale Assessing

There was no difference in the sleep structure before and after stimulation, including sleep efficiency, REM latency, sleep latency, total sleep time, and REM/N1/N2/N3 percentage (Table 2) in the active and sham groups.

**TABLE 2:** Polysomnography (PSG) sleep structure before and after treatment in tDCS and sham group.

	Before treatment	After treatment	*P*	95% CI
**Active stimulation**				
Sleep onset latency (SOL), min	27.03 ± 25.48	20.38 ± 21.12	0.387	[14.97, 45.76]
Total sleep time (TST),min	470.92 ± 56.44	483.22 ± 52.41	0.491	[428.94, 500.61]
Apnea hypopnea index (AHI)	6.36 ± 6.73	4.92 ± 5.22	0.465	[3.12, 11.05]
Stage 1 sleep ratio	15.67 ± 7.56	16.5 ± 9.71	0.770	[9.52, 20.97]
Stage 2 sleep ratio	66.8 ± 11.45	63.53 ± 11.03	0.376	[61.04, 75.84]
Stage 3 sleep ratio	4.48 ± 6.8	5.11 ± 7.24	0.785	[−0.45, 8.79]
REM sleep ratio	13.05 ± 9.94	14.86 ± 9.43	0.567	[5.77, 18.52]
REM latency, min	285.41 ± 131.09	244.5 ± 99.59	0.308	[226.03, 386.87]
**Sham stimulation**				
Sleep onset latency (SOL), min	47.89 ± 54.94	32.98 ± 32.67	0.343	[23.76, 86.92]
Total sleep time (TST), min	472.99 ± 71.04	484.41 ± 63.68	0.625	[420.14, 514.41]
Apnea hypopnea index (AHI)	9.04 ± 7.6	8.38 ± 10.4	0.836	[3, 15.73]
Stage 1 sleep ratio	15.12 ± 11.53	16.33 ± 10.17	0.748	[6.93, 22.12]
Stage 2 sleep ratio	67.16 ± 14.2	66.42 ± 9.45	0.860	[59.1, 75.95]
Stage 3 sleep ratio	3.38 ± 6.02	3.95 ± 5.5	0.775	[−0.94, 7.12]
REM sleep ratio	14.36 ± 7.92	13.29 ± 7.58	0.690	[9.48, 20.32]
REM latency, min	288.46 ± 100.37	283.94 ± 97.95	0.895	[221.43, 360]

*There were no statistically significant differences before and after treatment in two group.*

After the stimulation, there was a significant difference (*p* < 0.05) between the active and sham groups in the HRDS-24 early awakening scores. Active tDCS improves early awakening in depressed patients. In contrast, before the stimulation, there was no difference between the active and sham groups in the HRDS-24 early awakening scores (Table 3).

**TABLE 3:** Changes in hamilton depression rating scale total score and each factor score from baseline to after treatment.

Parameter	Baseline					After 10-session tDCS				
	Active group	Sham group	*p*	95% CI	Test	Active group	Sham group	*p*	95% CI	Test
Depressed mood	2.05 ± 1.13	3.17 ± 0.79	0.001*	[0.82, 2.13]	*t*-test	0.89 ± 0.81	0.83 ± 1.15	0.851	[0.26, 1.58]	*t*-test
Guilt	0.63 ± 0.83	1 ± 1.14	0.266	[−0.22, 1.1]	*t*-test	0.26 ± 0.45	0.22 ± 0.43	0.779	[−0.01, 0.58]	*t*-test
Suicide	0.63 ± 1.12	0.78 ± 1.26	0.711	[−0.24, 1.35]	*t*-test	0.21 ± 0.71	0.28 ± 0.75	0.782	[−0.32, 0.66]	*t*-test
Insomnia early	0.79 ± 0.85	1.06 ± 0.94	0.373	[0.06, 1.25]	*t*-test	0.26 ± 0.45	0.44 ± 0.78	0.392	[−0.26, 0.59]	*t*-test
Insomnia middle	0.79 ± 0.71	0.67 ± 0.59	0.574	[0.41, 1.29]	*t*-test	0.26 ± 0.45	0.28 ± 0.57	0.932	[−0.09, 0.6]	*t*-test
Insomnia late	0.63 ± 0.83	0.83 ± 0.86	0.472	[−0.04, 1.09]	*t*-test	0.16 ± 0.37	0.78 ± 0.94	0.030*	–	Rank sum test
Work and activities	1.95 ± 1.39	2.78 ± 1	0.046*	[0.71, 2.33]	*t*-test	0.79 ± 1.23	0.72 ± 1.07	0.861	[0.06, 1.6]	*t*-test
Retardation	0.37 ± 0.6	0.67 ± 0.77	0.217	–	Rank sum test	0.26 ± 0.45	0.5 ± 0.71	0.335	–	Rank sum test
Agitation	0.47 ± 0.7	0.67 ± 0.84	0.522	–	Rank sum test	0.21 ± 0.54	0.39 ± 0.78	0.420	[−0.32, 0.57]	*t*-test
Anxiety psychic	1.16 ± 0.9	1.83 ± 1.15	0.054	[0.13, 1.5]	*t*-test	0.68 ± 0.95	0.44 ± 0.78	0.408	[0.23, 1.39]	*t*-test
Anxiety somatic	1.89 ± 1.15	2.5 ± 1.15	0.119	[0.82, 2.35]	*t*-test	0.74 ± 0.87	0.94 ± 1	0.504	[0.01, 1.26]	*t*-test
Loss of appetite	0.32 ± 0.48	0.56 ± 0.62	0.193	[−0.18, 0.56]	*t*-test	0.21 ± 0.42	0.17 ± 0.51	0.777	[−0.08, 0.55]	*t*-test
Somatic symptoms	0.84 ± 0.83	1.28 ± 0.75	0.105	[0.08, 1.15]	*t*-test	0.21 ± 0.42	0.39 ± 0.5	0.250	–	Rank sum test
Sexual interest	0.11 ± 0.46	0.44 ± 0.92	0.162	[−0.55, 0.41]	*t*-test	0 ± 0	0.28 ± 0.83	0.152	[−0.52, 0.25]	*t*-test
Hypochondriasis	0.95 ± 1.03	0.78 ± 0.81	0.581	[0.41, 1.65]	*t*-test	0.16 ± 0.37	0.33 ± 0.59	0.287	[−0.27, 0.39]	*t*- test
Loss of weight	0.16 ± 0.5	0.28 ± 0.67	0.540	[−0.29, 0.49]	*t*-test	0.11 ± 0.46	0 ± 0	0.337	[−0.06, 0.37]	*t*-test
Insight	0.26 ± 0.45	0.39 ± 0.5	0.431	–	Rank sum test	0.21 ± 0.42	0.28 ± 0.46	0.645	[−0.12, 0.47]	*t*-test
Day–night change	1.16 ± 0.9	0.78 ± 0.88	0.202	[0.76, 1.94]	*t*-test	0.47 ± 0.7	0.33 ± 0.49	0.676	–	Rank sum test
Dispersonalization	0 ± 0	0 ± 0	–	–	–	0 ± 0	0 ± 0	–	–	–
Paranoid symptoms	0.11 ± 0.32	0.06 ± 0.24	0.592	[−0.06, 0.32]	*t*-test	0 ± 0	0 ± 0	–	–	–
Obsessive-compulsive	0.42 ± 0.77	0.33 ± 0.69	0.717	[−0.02, 0.95]	*t*-test	0.21 ± 0.54	0.11 ± 0.47	0.554	[−0.08, 0.6]	*t*-test
Helplessness	1.58 ± 0.61	1.56 ± 0.7	0.914	[1.16, 2.03]	*t*-test	0.68 ± 0.58	0.94 ± 0.94	0.315	[0.03, 1.07]	*t*-test
Hopelessness	0.84 ± 0.9	0.78 ± 0.65	0.805	[0.35, 1.4]	*t*-test	0.47 ± 0.51	0.61 ± 1.14	0.637	[−0.18, 0.99]	*t*-test
Self-abasement	1.21 ± 1.08	1.94 ± 1.35	0.076	[0.02, 1.65]	*t*-test	0.42 ± 0.77	0.61 ± 0.78	0.351	–	Rank sum test
Total	19.42 ± 7.33	25.11 ± 6.43	0.017*	[11.89, 21.11]	*t*-test	11.37 ± 16.28	9.83 ± 9.53	0.730	[3.18, 21.13]	*t*-test

The t-test and rank-sum test were applied for statistical analysis for data with Gaussian and non-Gaussian distributions. Any significant change from baseline (p-value<0.05) is in bold and starred. “*” means that significant change from baseline (p < 0.05).

After the stimulation, there were significant difference (*P* < 0.05) before and after stimulation, including depressed mood, early, middle, and late insomnia, work and activities, somatic anxiety, somatic symptoms, hypochondriasis, day-night change, helplessness, and self-abasement in active groups (Table 4). There were significant differences (*P* < 0.05) before and after stimulation, including depressed mood, guilt, early insomnia, work and activities, somatic anxiety, somatic symptoms, helplessness, self-abasement, and total scores in the sham group (Table 4).

**TABLE 4:** Changes in hamilton depression rating scale total score and each factor score before and after treatment in active-group and sham-group.

Parameter	Active-group				Sham-group			
	Baseline	After 10-session tDCS	*P*	95% CI	Baseline	After 10-session tDCS	*P*	95% CI
Depressed mood	2.05 ± 1.13	0.89 ± 0.81	0.001*	[1.98, 3.27]	3.17 ± 0.79	0.83 ± 1.15	0.000*	[3.67, 5]
Guilt	0.63 ± 0.83	0.26 ± 0.45	0.098	[0.38, 1.26]	1 ± 1.14	0.22 ± 0.43	0.010*	[0.81, 1.97]
Suicide	0.63 ± 1.12	0.21 ± 0.71	0.174	[0.22, 1.46]	0.78 ± 1.26	0.28 ± 0.75	0.158	[0.33, 1.73]
Insomnia early	0.79 ± 0.85	0.26 ± 0.45	0.023*	[0.61, 1.51]	1.06 ± 0.94	0.44 ± 0.78	0.041*	[0.78, 1.95]
Insomnia middle	0.79 ± 0.71	0.26 ± 0.45	0.010*	[0.66, 1.45]	0.67 ± 0.59	0.28 ± 0.57	0.054	[0.46, 1.25]
Insomnia late	0.63 ± 0.83	0.16 ± 0.37	0.030*	[0.44, 1.29]	0.83 ± 0.86	0.78 ± 0.94	0.854	[0.26, 1.48]
Work and activities	1.95 ± 1.39	0.79 ± 1.23	0.010*	[1.66, 3.39]	2.78 ± 1	0.72 ± 1.07	0.000*	[3.1, 4.51]
Retardation	0.37 ± 0.6	0.26 ± 0.45	0.544	[0.08, 0.77]	0.67 ± 0.77	0.5 ± 0.71	0.502	[0.25, 1.25]
Agitation	0.47 ± 0.7	0.21 ± 0.54	0.200	[0.19, 1.01]	0.67 ± 0.84	0.39 ± 0.78	0.311	[0.26, 1.36]
Anxiety psychic	1.16 ± 0.9	0.68 ± 0.95	0.122	[0.79, 2]	1.83 ± 1.15	0.44 ± 0.78	0.000*	[1.86, 3.2]
Anxiety somatic	1.89 ± 1.15	0.74 ± 0.87	0.001*	[1.81, 3.15]	2.5 ± 1.15	0.94 ± 1	0.000*	[2.55, 4.01]
Loss of appetite	0.32 ± 0.48	0.21 ± 0.42	0.475	[0.07, 0.66]	0.56 ± 0.62	0.17 ± 0.51	0.047*	[0.36, 1.13]
Somatic symptoms	0.84 ± 0.83	0.21 ± 0.42	0.006*	[0.73, 1.6]	1.28 ± 0.75	0.39 ± 0.5	0.000*	[1.29, 2.15]
Sexual interest	0.11 ± 0.46	0 ± 0	0.324	[−0.06, 0.37]	0.44 ± 0.92	0.28 ± 0.83	0.572	[−0.07, 1.12]
Hypochondriasis	0.95 ± 1.03	0.16 ± 0.37	0.003*	[0.83, 1.85]	0.78 ± 0.81	0.33 ± 0.59	0.069	[0.52, 1.49]
Loss of weight	0.16 ± 0.5	0.11 ± 0.46	0.738	[−0.13, 0.5]	0.28 ± 0.67	0 ± 0	0.087	[0.1, 0.74]
Insight	0.26 ± 0.45	0.21 ± 0.42	0.712	[0.01, 0.58]	0.39 ± 0.5	0.28 ± 0.46	0.494	[0.11, 0.77]
Day–night change	1.16 ± 0.9	0.47 ± 0.7	0.013*	[0.98, 2.03]	0.78 ± 0.88	0.33 ± 0.49	0.069	[0.52, 1.49]
Dispersonalization	0 ± 0	0 ± 0	–	[0, 0]	0 ± 0	0 ± 0	–	[0, 0]
Paranoid symptoms	0.11 ± 0.32	0 ± 0	0.154	[0.01, 0.3]	0.06 ± 0.24	0 ± 0	0.324	[−0.03, 0.2]
Obsessive-compulsive	0.42 ± 0.77	0.21 ± 0.54	0.334	[0.09, 0.97]	0.33 ± 0.69	0.11 ± 0.47	0.265	[0.04, 0.84]
Helplessness	1.58 ± 0.61	0.68 ± 0.58	0.000*	[1.63, 2.42]	1.56 ± 0.7	0.94 ± 0.94	0.029*	Rank sum
Hopelessness	0.84 ± 0.9	0.47 ± 0.51	0.129	[0.55, 1.51]	0.78 ± 0.65	0.61 ± 1.14	0.594	[0.23, 1.49]
Self-abasement	1.21 ± 1.08	0.42 ± 0.77	0.014*	[0.99, 2.23]	1.94 ± 1.35	0.61 ± 0.78	0.001*	[1.87, 3.36]
Total	19.42 ± 7.33	11.37 ± 16.28	0.057	[15.13, 31.75]	25.11 ± 6.43	9.83 ± 9.53	0.000*	[27.24, 38.26]

The t-test and rank-sum test were applied for statistical analysis for data with gaussian and non-gaussian distributions. Any significant change from baseline (p-value<0.05) is in bold and starred. “*” means that significant change from baseline (p < 0.05).

### Complexity

**FIGURE 4:**
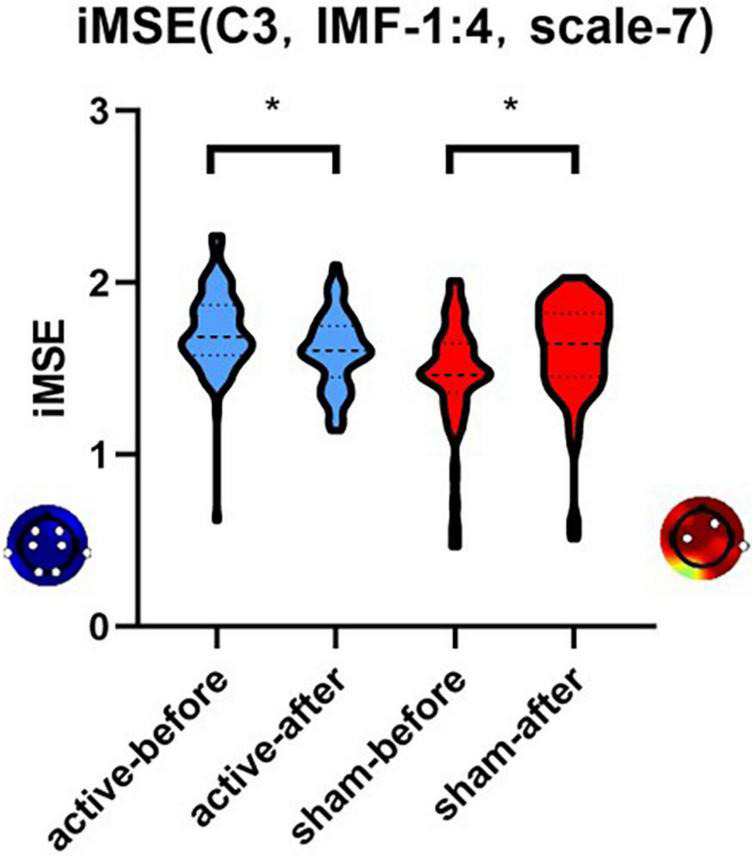
The differences in variation trend of iMSE in the case of channel C3, IMF 1:4, and scale 7. There was a different variation trend in the iMSE between the active and sham groups. “*” means that significant change from baseline (p < 0.05). Red colors represent increased EEG complexity and blue colors represent decreased EEG complexity.

After active stimulation there was a significantly decreased iMSE compared with pre-treatment for the REM stage (Figure 2). In addition, after sham stimulation there was a significantly increased iMSE compared with pretreatment (Figure 3). In Figures 2, 3, the difference in EEG complexity is reflected by multiple dimensions with different channels, IMFs, and scales in the same figure. For a better demonstration of our findings, the typical result of a single channel, IMF, and scale is necessary, which have been marked in Figures 2, 3 with a red frame. Typically, the differences in variation trend are reflected in channel C3, IMF 1:4, and scale 7 (Figure 4).

### Harms

Due to the lack of an appropriate scale for evaluating adverse effects of tDCS, only an open-ended survey was conducted in this study. Two patients in the active group complained of headaches. There were no corresponding reports in the sham group.

## Discussion

In the current study iMSE was used for the first time to analyze the efficacy of tDCS in treating sleep EEG complexity in patients with depression. We showed that the complexity of REM EEG signals significantly decreased iMSE after tDCS active stimulation but the complexity of REM EEG signals significantly increased iMSE in tDCS sham group.

The typical sleep structure is divided into RME and NREM sleep; NREM sleep is divided into N1, N2, and N3 (40). The N3 sleep stage represents the deepest sleep, which is characterized on EEG by high amplitude slow delta waves, and therefore is frequently referred to delta sleep or slow wave sleep (SWS) (3). Several studies have shown that increased SWS during NREM improved sleep quality and enhances memory consolidation (41). SWS reduction is closely related to anxiety and depression (42).

Funk et al. showed that slow waves also occurred during REM (43). A study involving the EEG microstructure of REM sleep divided REM sleep into phasic and tonic REM (43, 44). Phasic REM is characterized by SWS, and EEG frequency is mainly in the delta-theta range between 2 and 8 Hz, while the EEG frequency of tonic REM sleep is mainly in gamma >32 Hz. Based on sleep EEG analysis, increased theta activity decreased EEG complexity (45). Studies on the sleep mechanism showed that increased theta activity contributed to enhancing emotional memory, which might be the sleep-related mechanism underlying tDCS in improving depression (46). In 2016 Mariani et al. (47) reported an inverse relationship between nighttime sleep quality and EEG complexity. Terzano et al. (48) found a negative correlation between sleep EEG complexity and deep sleep.

Entropy is a common non-linear feature of EEG that indicates the complexity of the EEG signal. Typically, the high complexity of sleep EEG reveals poor sleep quality. As one of the methods to measure EEG complexity, MSE has been used in relevant clinical studies. In 2010 Takahashi et al. (49) used MSE to measure cortical abnormalities and intervention effects in schizophrenia. In 2013 Okazaki et al. (50) used MSE to analyze EEG before and after electroconvulsive therapy for depression. The EEG complexity of all patients decreased after ECT treatment. In 2015, Kuo et al. (51). evaluated the sleep quality of 32 adults based on the sleep EEG MSE. Kuo et al. (51) reported that the average MSE values in the poor sleep efficiency group was higher than the good efficiency group. In our work, iMSE was applied, and we showed that REM sleep EEG complexity decreased during active tDCS stimulation, revealing that iMSE is a sensitive measure of sleep EEG complexity.

The iMSE was applied in this study fit for non-stationary and non-linear EEG signals. We found that EEG complexity decreased significantly during REM in patients with depression after active tDCS treatment, but increased in patients with sham tDCS treatment. The increase in EEG complexity during the REM period in the control group may have been related to antidepressant treatment. The common point of different antidepressant actions involved positive modulation of 5-HT and NE systems in the central nervous system. These neurotransmitters, mainly derived from the dorsal raphe and LC, respectively, inhibit cholinergic REM-on neurons in the LDT/PPT and lead to REM-off and arousal (52). This mechanism may account for the REM inhibitory effect of most antidepressants, which may cause sleep disorders and sleep fragmentation. For the sham-tDCS group, the reason for increased REM EEG complexity is likely related to antidepressants. At the same time, tDCS antagonizes the effect and improves sleep quality at night in patients with depression.

In the current study we showed that the HDRS-24 early awakening factor score in patients treated with active tDCS was significantly lower than the control group, suggesting that tDCS improved early awakening symptoms in patients with MDD. In both the treatment group and the control group, the scores of multiple factors of HAMD-24 decreased before and after treatment, which was considered to be related to the continued effect of the combined therapy. Meta-analyses suggest some efficacy of tDCS in the treatment of acute depression disorder with moderate effect size, and low efficacy in treatment-resistant depression (16). The subjects in this study were mainly inpatients with relatively severe depressive symptoms, and the addition of tDCS showed no significant effect on clinical manifestations of depression and the reduction of *HDRS-24* score.

Previous studies have shown that tDCS treatment improved sleep quality with tDCS treatment between 9:00 a.m. and 12:00 a.m. during the day or night (53–56). The tDCS studies in healthy populations and patients with insomnia produced inconsistent results. Marshall et al. reported that tDCS in sleep time improved slow-wave sleep in healthy subjects (13). In 2019 Frase et al. showed that tDCS in sleep time increased nighttime arousal in patients with insomnia, which had been associated with a decrease in arousal threshold in insomniacs (15). By treating tDCS during the daytime, we not only made it more practical in the clinic, but also avoided the arousal threshold problem.

Our results suggested that daytime tDCS improved nighttime REM sleep EEG complexity in patients with MDD and iMSE, an effective and sensitive measure of assessing sleep quality by changes the EEG complexity during night sleep.

There were three major limitations of our work. First, the REM period was not separated as first, second, and third REM for analysis, which will be studied in the future. Second, after 2 weeks of fixed drug treatment, the intervention of drugs in treatment has been reduced as much as possible, however, the use of a single antidepressant treatment is challenging. The sample size shall be expanded continually to analyze drug influence in separate groups. Third, beside items of insomnia symptoms in HRDS-24, there was no information regarding self-reported sleep quality in this study, We will add the sleep self-rating scale in corollary study.

## Data Availability Statement

The original contributions presented in the study are included in the article/supplementary material, further inquiries can be directed to the corresponding authors.

## Ethics Statement

The studies involving human participants were reviewed and approved by the Ethics Committee of Suzhou Guangji Hospital. The patients/participants provided their written informed consent to participate in this study.

## Author Contributions

ZL mainly wrote this manuscript. XuZ and LF completed the evaluation of the experimental scale. YZ, WP, MY, YY, XF, and GL collected clinical data. YL and SG performed PSG analysis. NH was the originator of iMSE analysis methods and guided the explanation of the rationality of the methods. XiZ, XD, and RC helped in writing the manuscript. All authors have read and approved the final manuscript.

## Conflict of Interest

The authors declare that the research was conducted in the absence of any commercial or financial relationships that could be construed as a potential conflict of interest.

## Publisher’s Note

All claims expressed in this article are solely those of the authors and do not necessarily represent those of their affiliated organizations, or those of the publisher, the editors and the reviewers. Any product that may be evaluated in this article, or claim that may be made by its manufacturer, is not guaranteed or endorsed by the publisher.
